# Biochanin A attenuates myocardial ischemia/reperfusion injury through the TLR4/NF-κB/NLRP3 signaling pathway^1^

**DOI:** 10.1590/s0102-865020190110000004

**Published:** 2019-12-20

**Authors:** Yejun Bai, Zhigang Li, Weihao Liu, Dong Gao, Min Liu, Peiying Zhang

**Affiliations:** I Physician, First School of Clinical Medicine, Nanjing University of Chinese Medicine, and Department of Cardiovascular, Haimen Branch of Shanghai First People's Hospital Haimen Traditional Chinese Medicine Hospital, China. Conception and design of the study, manuscript writing.; Shanghai First People's Hospital Haimen Traditional Chinese Medicine Hospital, Department of Cardiovascular, China; II Physician, Department of Cardiovascular, Xuzhou Central Hospital Affiliated to Nanjing University of Chinese Medicine, China. Acquisition and analysis of data.; III Chief physician, Department of Cardiovascular, Xuzhou City Hospital of TCM Affiliated to Nanjing University of Chinese Medicine, China. Technical procedures, acquisition of data.; IV Chief physician, Department of Cardiovascular, Xuzhou Central Hospital Affiliated to Nanjing University of Chinese Medicine, China. Design and supervised all phases of the study.

**Keywords:** Biochanin A, Myocardial Reperfusion Injury, Inflammation, Toll-Like Receptor 4, Rats

## Abstract

**Purpose:**

Myocardial ischemia/reperfusion (Ml/R) injury is a leading cause of damage in cardiac tissues, with high rates of mortality and disability. Biochanin A (BCA) is a main constituent of Trifolium pratense L. This study was intended to explore the effect of BCA on Ml/R injury and explore the potential mechanism.

**Methods:**

*In vivo* MI/R injury was established by transient coronary ligation in Sprague-Dawley rats. Triphenyltetrazolium chloride staining (TTC) was used to measure myocardial infarct size. ELISA assay was employed to evaluate the levels of myocardial enzyme and inflammatory cytokines. Western blot assay was conducted to detect related protein levels in myocardial tissues.

**Results:**

BCA significantly ameliorated myocardial infarction area, reduced the release of myocardial enzyme levels including aspartate transaminase (AST), creatine kinase (CK-MB) and lactic dehydrogenase (LDH). It also decreased the production of inflammatory cytokines (IL-1β, IL-18, IL-6 and TNF-α) in serum of Ml/R rats. Further mechanism studies demonstrated that BCA inhibited inflammatory reaction through blocking TLR4/NF-kB/NLRP3 signaling pathway.

**Conclusion:**

The present study is the first evidence demonstrating that BCA attenuated Ml/R injury through suppressing TLR4/NF-kB/NLRP3 signaling pathway-mediated anti-inflammation pathway.

## Introduction

Myocardial ischemia-reperfusion (MI/R) injury refers to the aggravation of myocardial tissue injury or even permanent irreversible injury after blood flow is resupplied due to myocardial ischemia injury^1^. It has become one of the cardiovascular diseases with the highest morbidity and mortality in developed and developing countries^2^. Since MI/R injury was firstly reported by Jennings, it has been a research hotspot in cardiovascular diseases^3^. Despite the achievement in new treatments (thrombolysis, percutaneous revascularization^4^, percutaneous coronary intervention, bypass, etc.), there is still no way to completely prevent the additional damage caused by reperfusion itself. Basic studies have found that the pathological mechanism of MI/R is aerobic free radical injury, intracellular pH change, calcium overload, inflammatory factor infiltration, etc. In addition, other factors include immune imbalance, endoplasmic reticulum stress, apoptosis and autophagy, myocardial energy metabolism disorder, myocardial microvascular endothelial cell injury and so on.

Among them, myocardial inflammation has been recognized as an important hallmark of MI/R injury. Accumulating evidences have demonstrated that a large number of inflammatory mediators and chemokines are produced during I/R injury, and activated leukocytes, platelets, and vascular endothelial cells to express a large number of adhesion molecules, such as selectin and integrin, to promote adhesion of leukocytes to vascular endothelial cells and the accumulation of white blood cells in the blood vessels^5^. Simultaneously activated neutrophils can secrete cytokines such as TNF-α, IL-1β, and IL-6^6^ ^,^ ^7^. These cytokines play important roles in cell damage, such as inducing cell apoptosis^8^, the inflammatory response then continues to expand, causing further damage to the cardiomyocytes. Inflammatory reaction exposes vascular endothelial cell adhesion molecules, causing inflammatory cells to pass through the blood vessel wall, increasing the infiltration of inflammatory cells into vascular endothelium, leading edema of the vascular lumen caused by tissue edema, promoting microcirculatory disorders, further aggravating myocardial damage. Numerous studies have shown that anti-inflammatory effects can improve the progress of MI/R^9^. Naturally, strategies aimed at inhibiting inflammatory response are beneficial to ameliorate MI/R injury. Therefore, to explore and elucidate the protective mechanisms are urgent for new therapies of prevention of MI/R injury.

Biochanin A (5, 7-Dihydrox-4’-methoxyisoflavone, abbreviated as BCA) is the main effective component of *Trifolium pratense L.*, which has estrogen-like characteristics and is mainly used in the treatment of menopausal syndrome in women^10^ ^,^ ^11^. It has been revealed that BCA has a variety of pharmacological effects, such as anti-tumor, anti-inflammatory, anti-osteoporosis, antibacterial, hypolipidemic, etc^12^ ^–^ ^16^. Previous studies have proved that BCA can inhibit inflammatory responses in a variety of diseases. However, the cardioprotective effect of BCA and the underlying mechanism remains unclear. Wang *et al*.^17^ demonstrated that BCA protects rats from focal cerebral I/R by inhibiting p38-mediated inflammatory reaction. In addition, BCA has also been shown to protect acute liver injury and acute kidney injury by reducing inflammatory burden^18^ ^–^ ^20^. Notably, BCA was able to reduce inflammatory injury and neuronal apoptosis after subarachnoid hemorrhage by inhibiting the TLRs/TIRAP/MyD88/NF-κB Pathway^21^. We thus speculated that the inflammatory response mediated by the TLR4/NF-kB/NLRP3 inflammasome pathway may be involved in the regulation of BCA-mediated myocardial protection. Therefore, this study was designed to evaluate the role of BCA in preventing MI/R injury and to explore its possible mechanisms.

## Methods

All procedures were performed in accordance with the Guide for the Care and Use of Laboratory Animals published by the U.S. National Institutes of Health (NIH publication NO. 85-23, revised in 1996). The protocol was reviewed and approved by the Ethics Committee (Protocol number: SCXK (SU) 2016-0004).

A total of 30 adult male Sprague-Dawley (SD) rats (body weight 220–250g), obtained from the animal center of Animal Medicine Center, Xuzhou Medical University were used. All rats were maintained in diurnal lighting conditions (12 h/12 h) at a constant temperature of 25 ± 2°C for 7 days before experiment.

### Reagents

Biochanin A (BCA, with purity of 98%) was purchased from Shanghai Maclean Biochemical Technology Co., Ltd. (China). Antibodies against TLR4, MyD88, NF-κB, p-NF-κB, IkBα, p-IkBα, NLRP3, ASC, Caspase-1, GAPDH and the goat anti-rabbit secondary antibody were purchased from Abcam (Cambridge, MA, UK).

### In vivo MI/R model and treatment

The MI/R injury model was established as previously described^22^ ^,^ ^23^. In brief, rat model of MI/R injury was established by ligation of left anterior descending coronary artery for 0.5 h and reperfusion for 2 h. Sham-operation group was not ligated. Rats were randomly assigned to the following groups (6 rats in each group): (1) Sham group; (2) MI/R group; (3) MI/R + BCA (12.5mg/kg) group; (4) MI/R + BCA (25mg/kg) group; and (5) MI/R + BCA (50mg/kg) group. BCA was intragastrically administered every day for 7 days before operation (diluted in sterile saline containing less than 1% dimethyl sulfoxide (DMSO)). Finally, the hearts and blood samples from the sacrificed rats were then harvested for further pathological and biochemical analysis.

### Myocardial infarct size measurement

To investigate the effect of BCA on MI/R injury, the myocardial infarction areas in each group were measured by triphenyltetrazolium chloride (TTC) staining. The heart was stored at −18°C for 15 minutes and cut into 5 pieces equably at the line which was parallel to the coronary sulcus and below the heart ligature. All slices were weighedagain and incubated for 15 minutes in TTC for pathological examination at 37°C in the dark. An Image-Pro Plus image analysis software (Version 4.1, Media Cybernetics, LP, USA) was used to analyze non-TTC stained area (white or pale, infarct area) and TTC stained area (red, non-infarct area). Finally, the formula: Infarct size (%) = (infarct area/whole heart area) × 100% was used to calculate the proportion of infarcted myocardium to the entire myocardial tissue.

### Detection of myocardial enzyme levels in serum

Rats were sacrificed after peripheral blood was collected, serum was separated to evaluate heart muscle damage indicators, including aspartate transaminase (AST), creatine kinase (CK-MB) and lactic dehydrogenase (LDH), using ELISA kit (Thermo Fisher Scientific, USA).

### Detection of inflammatory cytokines in serum

The levels of IL-1β, IL-6, IL-18 and TNF-α in serum were analyzed by ELISA kits based on the manufacturer's Instructions. Then the optical density (OD) was read at 450 nm by a microplate spectrophotometer. Finally, the concentrations of the cytokines were calculated by reference to the standard curves.

### Western blot

Protein from tissues and cells were obtained and qualified with a BCA detecting kit (Beyotime). A total of 30 µg protein was separated by SDS-PAGE and electro-transferred onto PVDF membrane (Millipore, USA). After blocking with 5% BSA, the membrane was incubated overnight with primary antibody, such as anti-TLR4 (1:500, # ab13556), anti-MyD88 (1:500, # ab2064, Abcam), anti-NF-κB (1:1000, #ab16502, Abcam), anti-p-NF-κB (1:500, ab86299, Abcam), anti-IkBα (1:1000, #ab32518, Abcam), anti-p-IkBα (1:10000, #ab133462, Abcam), anti-NLRP3 (1:500, #ab214185, Abcam), anti-ASC (1:1000, #ab47092, Abcam), anti-Caspase-1 (1:1000, #ab207802, Abcam), and anti-GAPDH(1:2500, #ab9485, Abcam) at 4 °C for overnight. Then, the membranes were incubated with with HRP-conjugated goat anti-rabbit IgG (1:2000, #ab6721, Abcam) for 45 min at room temperature. Finally, the bands were examined with ECL reagent (Beyotime). The signals were analyzed via Image Lab™ Software (NIH Image, Bethesda, USA).

### Statistical analyses

The software GraphPad 7.0 was used for data analysis. All data were repeated at least three times and are shownn as mean ± standard deviation (SD). Student's *t*-test and one-way ANOVA were used to compare the signiﬁcance of differences among experimental groups. If *p*<0.05, the data were statistically significant.

## Results

### Effects of BCA on myocardial infarction area in MI/R rats

**Figure 1:**
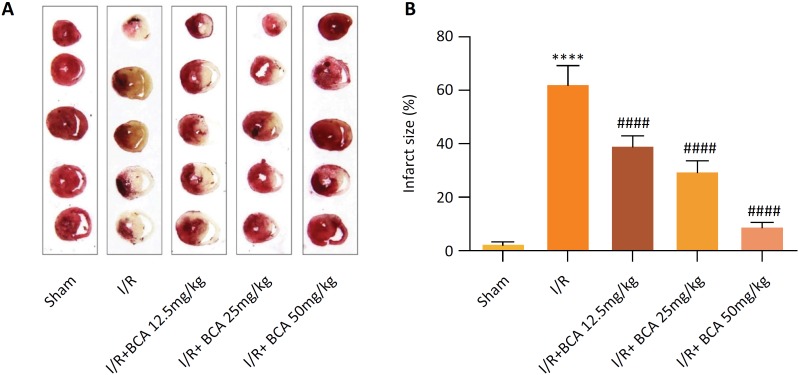
Effects of BCA on myocardial infarction area in MI/R rats. **(A)** Representative images of myocardial infarct size (n=6 in each group); Red stain–viable area; White stain-infarct region. **(B)** Quantitative analysis of infarct size proportions of myocardial tissues (n=6 in each group). ****p < 0.00001 compared with Sham group; ^####^ *p* < 0.00001 compared with MI/R group.

The results from TTC staining revealed that the infarct area in I/R group was increased sharply as compared to that in the sham group, confirming the successful construction of rat model of MI/R (*p*<0.0001). Meanwhile, BCA (12.5, 25 and 50 mg/kg) observably reversed the abnormal increased infarct size in rats subjected to MI/R injury in a dose-dependent manner (*p*<0.0001) (Fig. 1 A,B).

### Effects of BCA on myocardial enzyme markers in MI/R rats

**Figure 2:**
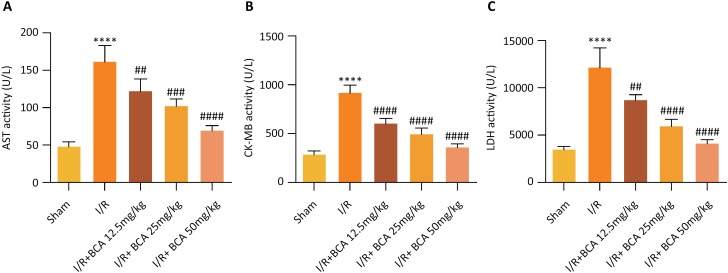
Effects of BCA on myocardial enzyme markers in MI/R rats **(A)** Serum aspartate transaminase (AST) levels. **(B)** Serum creatine kinase-MB (CK-MB) levels. **(C)** Serum lactic dehydrogenase (LDH) levels. n=6 in each group. ****p < 0.00001 compared with Sham group; ^##^ *p* < 0.01, ^###^ *p* < 0.001 and ^####^ *p* < 0.00001 compared with MI/R group.

AST, CK-MB and LDH are main indexes of myocardial injury as shown in Figure 2 A-C, .Significantly increased levels of AST, CK-MB and LDH in serum were observed in MI/R group compared to that in Sham group (*p*<0.0001). However, BCA treatment dramatically reduced the MI/R–induced levels of AST (p=0.0086, p=0.0005 and *p*<0.0001), CK-MB (*p*<0.0001, *p*<0.0001 and *p*<0.0001) and LDH (p=0.0030, *p*<0.0001 and *p*<0.0001) in a dose-dependent manner.

### Effects of BCA on serum inflammatory cytokines in MI/R rats

**Figure 3:**
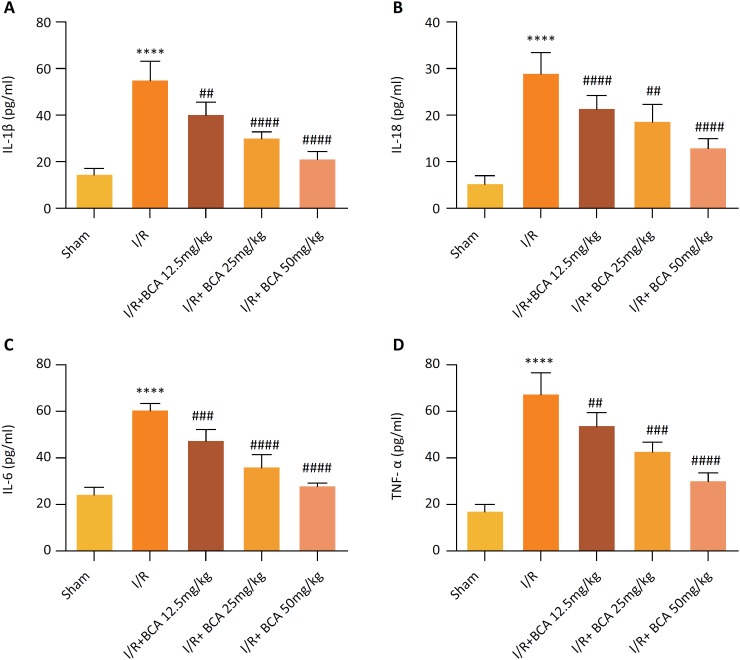
Effects of BCA on serum inflammatory cytokines in MI/R rats **(A)** Serum IL-1β levels. **(B)** Serum IL-18 levels. **(C)** Serum IL-6 levels. **(D)** Serum TNF-α levels. n=6 in each group. ****p < 0.00001 compared with Sham group; ^##^ *p* < 0.01, ^###^ *p* < 0.001 and ^####^ *p* < 0.00001 compared with MI/R group.

Inflammatory cytokines have been found to be key indicators of MI/R injury. As shown in Figure 3, there were noticeable enhancements in serum levels of IL-1β, IL-18, IL-6 and TNF-α in the MI/R group in contrast to the Sham group (*p*<0.0001). Inversely, treatment with BCA (12.5, 25 and 50 mg/kg) significantly reduced MI/R-induced inflammatory cytokines, IL-1β (*p*=0.0042, *p*<0.0001 and *p*<0.0001), IL-18(*p*<0.0001, *p*=0.0018 and *p*<0.0001), IL-6(*p*=0.0002, *p*<0.0001 and *p*<0.0001) and TNF-α (*p*=0.0119, p=0.0001 and *p*<0.0001), in a dose-dependent manner.

### Effects of BCA on the TLR4/ NF-κB signaling pathway in MI/R rats

**Figure 4:**
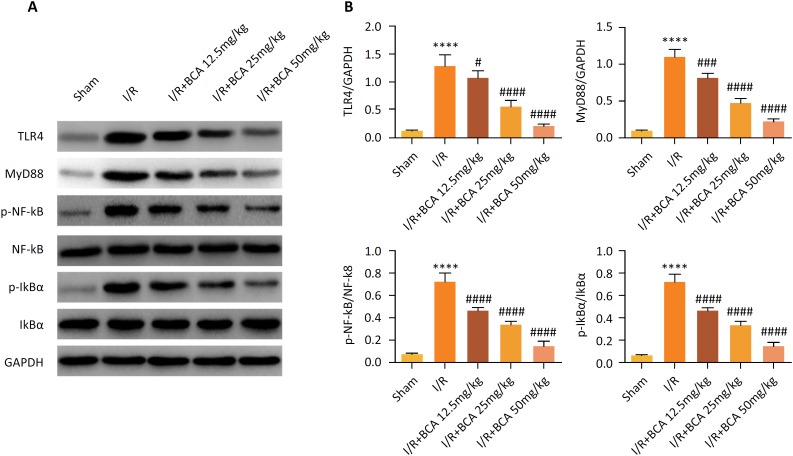
Effects of BCA on the TLR4/ NF-κB signaling pathway in MI/R rats **(A)** Representative images of the Western blot results. **(B)** Quantitative densitometric analysis of proteins. n=6 in each group. ****p < 0.00001 compared with Sham group; ^#^ *p* < 0.05, ^###^ *p* < 0.001 and ^####^ *p* < 0.00001 compared with MI/R group.

Considering the fact that TLR4/ NF-κB mediated inflammatory signaling pathway is associated with MI/R injury, the potential regulatory mechanism underlying the protective role of BCA in MI/R injury was then explored.. Western blot assay (Fig. 4 A–B) illustrated that TLR4, MyD88, p-NF-κB and p-IkBα were obviously increased in the MI/R group compared with the sham group (*p*<0.0001). Notably, the increased expression of TLR4 (*p*=0.0439, *p*<0.0001 and *p*<0.0001), MyD88 (*p*=0.0001, *p*<0.0001 and *p*<0.0001), p-NF-κB (*p*<0.0001, *p*<0.0001 and *p*<0.0001) and p-IkBα (*p*<0.0001, *p*<0.0001 and *p*<0.0001) induced by MI/R were significantly reversed by BCA (12.5, 25 and 50 mg/kg) treatment in a dose-dependent manner.

### Effects of BCA on the activation of NLRP3 inflammasome in MI/R rats

**Figure 5:**
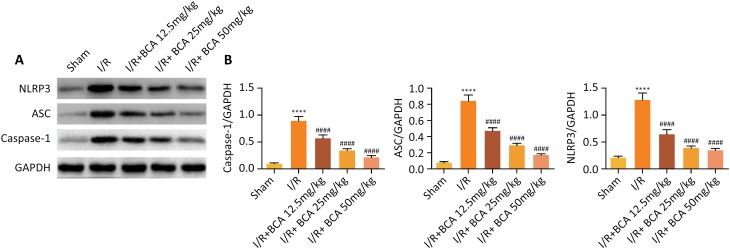
Effects of BCA on the activation of NLRP3 inflammasome in MI/R rats. **(A)** Representative images of the Western blot results. **(B)** Quantitative densitometric analysis of proteins. n=6 in each group. ****p < 0.00001 compared with Sham group and ^####^ *p* < 0.00001 compared with MI/R group.

Finally, we examined the level of NLRP3 inflammasome in the myocardium to clarify whether the anti-inflammatory effects of BCA are related to NLRP3 inflammasome. As depicted in Figure 5 A–B, MI/R injury significantly promotes up-regulation of NLRP3, ASC, and caspase-1 levels (*p*<0.0001). Whereas BCA (12.5, 25 and 50 mg/kg) treatment was effective in inhibiting the levels of NLRP3 (*p*<0.0001), ASC (*p*<0.0001), and caspase-1 (*p*<0.0001).

## Discussion

With the development of social economy and improvement of living standards, the risk factors of cardiovascular disease are increasing. Tissue damage caused by myocardial ischemia is an important cause of fatal diseases and is common in clinical practice. The pathophysiological process of myocardial I/R injury involves multiple mechanisms: inflammation, reactive oxygen species production, apoptosis, mitochondrial dysfunction, intracellular calcium overload, etc^26^ ^–^ ^28^. Therefore, exploring the complex pathological mechanism of reperfusion injury is expected to provide a promising therapeutic strategy for the treatment of MI/R injury.

In the present study, the infarct area in I/R group was increased sharply as compared to that in the sham group. These data proved that MI/R injury model was successfully established, and BCA significantly reduce myocardial infarction area in MI/R rats. BCA also suppressed the activities of main indexes of myocardial injury AST, CK-MB and LDH, indicating its capacity to ameliorate cardiac damage in MI/R rats. BCA could also suppress MI/R-induced inflammatory responses by decreasing the serum levels of IL-1β, IL-18, IL-6 and TNF-αin MI/R rats. This, based on the fact that the production of IL-1β and IL-18 not only requires TLR4-induced gene transcription, but also requires the activation of Caspase-1 by NLRP3 inflammasome, and Caspase-1 cleaves pro-IL-1β and pro-IL-18 to form the active forms of IL-1β and IL-18^25^. Thus, to explore the underlying mechanism, further analysis verified that BCA not only decreased the TLR4/ NF-κB signaling pathway, but also suppressed NLRP3 inflammasome in MI/R rats.

Damage to the myocardial cell membrane will lead to the release of myocardial enzymes and proteins, including AST, CK-MB and LDH, into the peripheral blood, which are widely used as reliable biomarkers to determine the degree of myocardial injury in clinic^29^. Results of the reduced myocardial infarct size and decreased levels of AST, CK-MB and LDH by BCA treatment compared to the MI/R group, together confirmed the protective effect of BCA on MI/R injury. MI/R injury is also related to the acceleration of inflammatory responses in cardiac tissue. Ischemia injury is caused by a temporary lack of blood supply to the tissue, while reperfusion injury following blood resupply is accompanied by a strong inflammatory response^30^. Therefore, reducing the release of inflammatory cytokines (IL-1β, IL-18, IL-6 and TNF-α) is an effective strategy to protect cardiac inflammation^31^ ^,^ ^32^. IL-1β and IL-18 are thought to be major triggers for the release of other inflammatory cytokines such as IL-6 and TNF-α^24^. Consistently, our data demonstrated that BCA treatment obviously inhibited the cardiac inflammation via inhibiting the production of inflammatory cytokines in MI/R rats.

It is well known that TLR4/NF-kB signaling pathway is involved in mediating inflammatory responses and the pathogenesis of MI/R injury through regulating pro-inflammatory cytokine production^25^ ^,^ ^30^ ^,^ ^33^. Activation of TLR4 promotes elevation of NF-kB, and NF-kB regulates expression of pro-inflammatory cytokines^34^. In addition, NLPR3 recruits in the heart during myocardial infarction and enhances the inflammatory response^35^. The NLRP3 inflammasome is a member of the Nod-like receptor (NLR) family and consists of a nucleoside-binding oligomeric domain-like receptor (NLRP3), a caspase recruitment domain (ASC), and caspase-1^36^. Substantial evidences have indicated that the NLRP3 protein is polymerized and bound to the ASC adapter to induce translocation and activation of caspase-1. In addition, activated caspase-1 is responsible for triggering the secretion of mature forms of secretions by pro-inflammatory cytokines. That is, activated Caspase-1 cleaves the precursors of IL-1β and IL-18 to form active IL-1β and IL-18, which are released into the extracellular region to recruit inflammatory cells to aggregate and expand the inflammatory response^37^. In the present study, BCA significantly reduced the serum production of IL-1β, IL-18, IL-6 and TNF-α, inhibited the expression levels of TLR4, MyD88, NLRP3, ASC, caspase-1 and the phosphorylations of IkB-α and NF-kB in MI/R rats. Taken together, these data revealed that BCA exerts its cardioprotective effects by inhibiting the inflammatory response through negatively regulating the TLR4/NF-κB/NLRP3 signaling pathway.

## Conclusion

Overall, the present study is the first evidence confirming the protective role of BCA in MI/R injury. More importantly, the TLR4/NF-κB/NLRP3 mediated inflammatory pathway might be served as the potential mechanism. Further detailed research is needed to explore the clinical application of BCA.
